# The Impact of COVID-19 on Maternal Health: Quantitative Data Related to Risk and Protective Factors Among Pregnant and Postpartum Women in Puerto Rico

**DOI:** 10.3390/ijerph22020141

**Published:** 2025-01-22

**Authors:** Irene Lafarga Previdi, Nobel Hernández Otero, Ana Guzzi Vasques, Ishwara Ayala, Génesis Alvelo Colón, Natacha Guilloty, Jessica Medina, Marielane Cancel-Garcia, José Cordero, Akram N. Alshawabkeh, Carmen Vélez Vega

**Affiliations:** 1 Center for Collaborative Research in Health Disparities, UPR Medical Sciences Campus, San Juan 00921, Puerto Rico; ana.vasques1@upr.edu (A.G.V.); carmen.velez2@upr.edu (C.V.V.); 2 Medical Sciences Campus, University of Puerto Rico, San Juan 00921, Puerto Rico; nobel.hernandez@upr.edu (N.H.O.); genesis.alvelo1@upr.edu (G.A.C.); marielane.cancel@upr.edu (M.C.-G.); 3 College of Engineering, Northeastern University, Boston, MA 02115, USA; ishwara.ayala@upr.edu (I.A.); natacha.guilloty@upr.edu (N.G.); medinalugardo@gmail.com (J.M.); a.alshawabkeh@northeastern.edu (A.N.A.); 4 College of Public Health, University of Georgia, Athens, GA 30602, USA; jcordero@uga.edu

**Keywords:** maternal health, COVID-19, health disparities, mental health

## Abstract

Background: The COVID-19 pandemic affected access to healthcare and social support. This especially impacted vulnerable populations like pregnant and postpartum women. Purpose: The specific aims of the project are the following: (1) examine the impact of COVID-19 on pregnancy experiences and outcomes; (2) examine the mental health impact of COVID-19 in pregnant women and mothers of children 12 months or younger; (3) identify risk and protective factors among this population in Puerto Rico. Methods: Participants were recruited from the Puerto Rico Testsite for Exploring Contamination Threats (PROTECT) cohort, which is composed of pregnant women and mothers from the northern karst region of Puerto Rico. This research has a mixed methods approach with a quantitative survey (n = 184) and qualitative interviews (n = 10); data collection was performed remotely. Findings: Results from the survey (n = 184) show that 20% of the cohort gave birth alone, 39% were separated from their baby after birth, 21% experienced isolation before birth, and 20% were separated after birth. In the study, 54% of the women were very worried about giving COVID-19 to their baby and avoided going out, receiving visits, and even canceling baby showers. The most reported sources of stress were their health status, work situation, and childcare, while the most reported coping mechanisms were watching TV or playing video games, using social media, and talking with loved ones. Forty-two percent reported that they frequently stopped enjoying activities that used to make them happy, and only 21% considered seeking mental health support. Conclusion: COVID-19 restrictions changed initial plans for baby showers, births, and childcare, and impacted the participants’ mental health. Physical distance measures have resulted in isolation and stress. We expect these findings to lead to developing interventions for community health centers and parents/caretakers in Puerto Rico.

## 1. Introduction

### 1.1. Maternal Health and Emergencies

According to the World Health Organization [1], maternal health “refers to the health of women during pregnancy, childbirth and the postnatal period”. Although there have been significant advances in the health of women and their children, there are instances that continue to challenge their well-being. For example, after a natural disaster such as a hurricane, it has been identified that a lack of food, water, and shelter adversely impacts pregnancy [2]. In addition, the literature indicates that women survivors of natural disasters are particularly vulnerable to suffering psychological consequences such as post-traumatic stress disorder (PTSD), depression, and intimate partner violence [3,4].

In the last decade in the archipelago of Puerto Rico, several emergencies and socio-natural disasters have affected maternal and child health. Hurricanes Irma and Maria in 2017 revealed that women in the pregnancy and postpartum period had significant challenges, such as unplanned c-sections, lack of access to essential services, loss of housing and work, concerns about access to health services, challenges in nutrition, and exposure to environmental damage [5,6]. In 2019, Puerto Rico was shaken by a series of earthquakes that disproportionately affected the island’s south. This event, like past emergencies, revealed once again the poverty, social inequities and inequalities in the country, the lack of social support for families and their children, as well as the absence of an organized and concerted response by the State to public health emergencies [7]. In 2020, a pandemic was declared due to COVID-19 [8]. Among the most vulnerable populations were pregnant women and children [9].

### 1.2. Mental Health, Pregnancy, and Postpartum

There is evidence in the literature on the impact of epidemic crises, social isolation, and restrictions in hospitals on the mental health and well-being of pregnant and postpartum women [10]. As a result of the crises associated with the COVID-19 pandemic, pregnant women had to deal with extra anxiety due to several factors, for example, the possibility of infection during pregnancy and/or after delivery, limited contact with family related to restrictions on delivery, and loss of social support due to quarantine and lockdown [10]. Also, school and daycare closures and the reduced availability of outside help have led to months of additional work for women. For working mothers, this has meant balancing full-time employment with childcare and schooling responsibilities. Furthermore, access to childcare was also a component that may have had an impact on women’s mental health related to COVID-19. The literature suggested that childcare closures due to COVID-19 were mostly concentrated in low-income and middle-income neighborhoods, and many families were not able to access childcare services, making it harder for parents to return to work [11].

### 1.3. Access to Healthcare Services

The quality of maternal and newborn care (QMNC) worldwide has been particularly affected by the pandemic, according to a study conducted in 12 countries of the WHO European region [12]. Some findings from the research conducted by Lazzerini and collaborators (2022) were that 62% of women had limitations regarding having a person to accompany them, 30.3% did not experience clear communication from health workers, and 49.9% of women perceived a reduction in QMNC due to the COVID-19 pandemic. Regarding maternal and perinatal care, according to Kotlar and collaborators (2021), who conducted a systematic literature review, the pandemic affects this indirectly and directly [13]. They found that, in general, prenatal and postnatal visits decreased due to the focus on the public health emergency. In addition, hospitals implemented policies that resulted in the isolation of pregnant women during the labor process. In Puerto Rico, the protocols implemented for the birthing process were based on government task force recommendations. From these recommendations, two central aspects can be highlighted: (1) the separation of the baby and mother when she was COVID-19 positive or considered a suspected case, and (2) the limitation of the number of people in the room. However, these recommendations were placed to prevent contagion in case of positive or suspected cases but were used in general practice by the healthcare system [14].

### 1.4. Significance

Pandemics and emergencies can affect access to medical and social services. According to the Centers for Disease Control (CDC), disasters like hurricanes or pandemics can present unique challenges to pregnant women and mothers of children 12 months old or younger, as well as potential exposures that can affect the developing fetus [15]. The findings can serve as a basis for future research projects regarding maternal health and emergency preparedness in Puerto Rico and also in the general U.S. Latino Hispanic population. They can serve to inform evidence-based protocols for individuals, communities, and healthcare settings to promote diverse strategies to manage a healthy pregnancy in emergency situations. The findings from this study can also be used to inform health policy or as a foundation for comparisons with experiences before/after the pandemic.

### 1.5. Specific Aims

Aim 1: Examine the impact of the COVID-19 outbreak on pregnancy-related experiences and outcomes.

Aim 2: Examine the mental health impact of the COVID-19 outbreak on pregnant women and mothers of children 12 months old or younger.

Aim 3: Identify risk and protective factors (related to mental health) of pregnant women and mothers of children 12 months old or younger in the COVID-19 outbreak.

## 2. Methods

### 2.1. Participants

**Figure 1:**
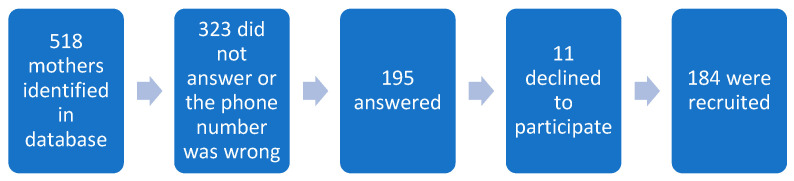
Flowchart of the recruitment process. In the research proposal, the desired sample number was 200 participants.

Participants were recruited from the PROTECT Center, which is composed of pregnant women and mothers of children 12 months old or younger from the North Karst Region of the archipelago of Puerto Rico. The PROTECT Center is a Superfund Research Program established in 2010 that studies the role of environmental contaminants on preterm births in Puerto Rico [16]. A total of 184 pregnant women and/or mothers from the PROTECT cohort were recruited and answered the survey virtually by phone between January 2021 and June 2022 (see Figure 1). The participants had delivered between late 2019 or during the year of 2020. Qualitative interviews were also conducted but this manuscript will focus on the quantitative survey.

### 2.2. Instrument

The survey instrument included the following sections: sociodemographic profile, socioeconomic and environmental factors, pregnancy experiences and outcomes, mental health and COVID-19 mitigation measures, including physical distancing, social support, healthcare access and services, and current employment situation. The socioeconomic and environmental factors included questions related to how the pandemic affected their economic situation and their access to healthy foods, the prevention measures they implemented, and their access to internet and transportation, among others. The social and emotional support sections consisted of questions related to the types of support they received and from which sources. The pregnancy experiences and outcomes section had questions related to how the pandemic changed their initial plans for pre- and postnatal care, and what kinds of adjustments they made to prevent COVID-19 contagion. The mental health perception section included questions related to sources of stress and stress management strategies as well as questions about if they felt they needed or did not need professional support during this time. The healthcare access section included questions related to the access to health-related services and how they would qualify the treatment from service providers. This was an original survey created for this study and is not a validated tool.

### 2.3. Recruitment Process and Data Collection

The participants were contacted by phone to explain the proposed study in detail and obtain informed consent. Once verbal consent was obtained, the research staff administered the survey by phone, annotated the participants’ responses, and uploaded the completed instrument with the participants’ ID and completion date to a Dropbox file. The retrospective aspect could have resulted in recall bias, but the experiences were relatively recent and participants were able to answer survey questions easily. After this step, the research staff coordinated the delivery of the monetary incentive and the informed consent document at one of the clinics affiliated with PROTECT. The IRB at the University of Puerto Rico, Medical Sciences Campus (Protocol No. B3300120) approved this study.

### 2.4. Data Analysis

The survey responses were collected using the ReDCap online data capture tool. Descriptive analyses were conducted to identify distributions, trends, and patterns related to pregnancy experiences and outcomes, mental health, sociodemographic characteristics, and socioeconomic factors. Crosstabulation analysis and Chi square tests, among variables of interest related to social determinants of health and health disparities, were performed. Stata program version 17 was used to realize analyses. This was an exploratory and descriptive study. The results will be used to describe and summarize the experiences of pregnant and postpartum women during the COVID-19 pandemic in Puerto Rico. The findings will serve to explain the specific aims of examining the impact of the pandemic on pregnancy and birth outcomes and on maternal mental health.

## 3. Results

### 3.1. Participant’s Demographic Profile

A total of 184 expectant mothers from the PROTECT cohort and/or mothers with infants under 1 year old were enrolled. Of the participants, 34.8% were 25 years old or younger and 45.1% of them lived in the municipalities of Camuy (15.8%), Arecibo (15.2%), and Bayamón (14.1%). Of the participants, 45.1% indicated that they were homemakers/stay-at-home mothers. Meanwhile, other professions that were mentioned by the participants were teacher (4.9%), nurse (4.3%), and cashier (3.8%), and some of them reported that they were unemployed (3.8%). In terms of annual income, 27.3% of the participants indicated they had an income of $10,000 or less, 26.8% reported an income between $10,000–$14,999, and 19.7% reported an income of $15,000–$24,999.

Of the participants, 81% indicated that they were “married” (44.0%) or were “living together” with their partner (37.0%). In addition, 61.5% of the participants had achieved a degree after graduating high school. Additionally, 93.4% of the participants were not pregnant when they completed the questionnaire, and 6.6% were pregnant. Out of the 6.6% that were pregnant, the median gestational age was 19 weeks. Also, 97.8% of the participants indicated that they have children, and when asked how many, 44.7% had one child, 41.9% had two children, and 12.8% had three children, with a median of two children. Regarding the number of people that live with them in the household, 39.9% of participants indicated four people.

### 3.2. Socioeconomic and Environmental Factors

Table 1 shows participants’ responses regarding specific health determinants in relation to COVID-19. When asked how COVID-19 affected their work, 31.5% of the participants indicated that the pandemic did not impact their work, while 15.8% reported that they did not have a job to begin with. On the other hand, participants reported that because of the COVID-19 pandemic, they either lost their jobs temporarily (12%) or lost their jobs permanently (10.3%). Similarly, when asked about the impact of the pandemic on their spouse’s job, they mentioned that their spouses lost their job temporarily (19.6%), lost their job permanently (7.6%), or had a decrease in job hours (10.9%).

In addition, 62.5% of participants mentioned that they did not face economic difficulties during this time. However, 47.8% indicated they needed some type of financial assistance, and when asked what type, the participants mentioned regular unemployment (50.0%) and Pandemic Unemployment Assistance (33.0%) as sources of support during the pandemic. In addition, 95.1% of the participants mentioned having access to nutritious and balanced meals, but 56.0% mentioned that they needed food assistance during the pandemic. When asked what type, the participants who had answered yes (n = 102) mentioned food coupons (80.2%) and food packages (40.6%).

Regarding access to essential services, almost all participants (97.8%) reported that they had access to a car for transportation. Regarding health services and insurance, almost all participants (98.4%) mentioned that they had health insurance; 63.5% had public insurance, and 36.5% had private insurance. Also, 91.8% of participants indicated that they had access to Wi-Fi and 73.2% had access to computers. In the same way, when asked which protective measures they implemented to prevent the contagion of COVID-19, almost all participants indicated the following: use of a face mask (100%), social distancing (99.5%), handwashing (98.9%), cleaning and disinfecting surfaces (96.7%), and the use of hand sanitizer (96.2%). Concerning COVID-19 exposure, 65% of participants reported that they had not had a family member diagnosed with COVID-19, and 96.7% stated that they had not had any family member die from the virus. When asked about whether a close family member became dependent due to the pandemic, physically (91.8%) or economically (97.3%), most answered no (see Table 1).

### 3.3. Social Support

Table 2 illustrates the distribution of various types of social support received by participants during the COVID-19 pandemic. When asked what type of social support the participants received during the pre- and postnatal period, the most reported were financial (40.8%), childcare (34.2%), and running errands (29.3%). Specifically, the sources of the social support received during this period were mostly their partner or spouse (65.8%), family members, especially parents (82.1%), and the government (25.5%) (see Table 3). When asked to rate the quality of the support received from different sources, the participants rated the support from family and neighbors (82.2%, n = 174) and community or religious organizations (55%, n = 20) as excellent. Meanwhile, they rated the government’s support quality (43.5%, n = 46) as good.

### 3.4. Pregnancy and Birth Experiences

During the COVID-19 pandemic period, 131 participants experienced childbirth. Table 4 outlines a comprehensive overview of their pregnancy and childbirth encounters amid the pandemic. Notably, 19.8% of participants underwent labor alone, while 21.4% were isolated before childbirth, and 38.9% were separated from their newborn after delivery. Furthermore, just over half of the participants (54.2%) expressed profound concerns about transmitting COVID-19 to their infant. The participants were specifically asked if they changed their birth plan as a consequence of the pandemic.

In order to prevent contagion, most of the participants reported that they engaged in the following prevention measures: avoided going out, avoided going to crowded places like restaurants and malls, canceled their baby showers, and avoided visits from other people who did not live with them (this included parents and other family members) (see Table 5). Of participants, 54.67% of those who received financial support ordered takeout or delivery as a preventive measure, compared to 18.35% of participants who did not receive financial support (*p* = 0.000). Of the participants who canceled their baby shower (n = 33), 33.33% received financial support (*p* = 0.000). Of the participants who canceled their baby shower (n = 33), 38.39% received support in performing their errands (*p* = 0.000).

### 3.5. Mental Health Perception

Table 6 shows the sources of stress related to the pandemic identified by the participants. The most reported stress sources were regarding the health status of themselves and their immediate family, the work situation of their partner in most cases, and the challenges related to childcare and distance learning. Of participants, 20.77% (n = 38) felt ‘worried about the future frequently and very frequently’ and 16.94% (n = 31) of participants ‘worried about the present frequently and very frequently’. Of participants, 68.68% indicated that they frequently or very frequently stopped enjoying activities that previously made them happy (n = 125). Of those worried about the future, 37.04% reported that caring for children had been one of their greatest sources of stress due to COVID-19 (*p* = 0.002). Of those worried about the future, 41.38% reported that social distancing had been one of their greatest sources of stress due to COVID-19 (*p* = 0.000). Of those worried about the future, 47.06% reported that the impact on the family had been one of their greatest sources of stress due to COVID-19 (*p* = 0.000). Of those participants worried about the present, 31.5% reported that taking care of children had been one of their greatest sources of stress due to COVID-19 (*p* = 0.003). Of those worried about the present, 31.03% reported that social distancing had been one of their greatest sources of stress due to COVID-19 (*p* = 0.001). Of those worried about the present, 35.29% reported that the impact on the family had been one of their greatest sources of stress due to COVID-19 (*p* = 0.000). The most reported coping mechanisms were watching TV or playing video games, using social media, and talking with loved ones. Of those participants who indicated that they frequently or very frequently stopped enjoying activities that previously made them happy, 78.2% did not consider seeking psychological support (*p* = 0.007).

### 3.6. Access to Healthcare Services

Despite the challenges presented by the pandemic, a vast majority of participants (98.5%, n = 130) were able to attend their prenatal appointments, ensuring uninterrupted continuity of care. Similarly, access to prenatal vitamins was nearly universal, with 97.69% of respondents reporting availability. Moreover, a significant portion of participants (86.3%) rated the treatment received from health professionals as good or excellent, reflecting the quality of care provided. When queried about other health conditions during both the pre- and postnatal periods, the majority (78.1%, n = 183) indicated that they did not experience any additional health issues.

## 4. Discussion

The findings from the survey reflect how the COVID-19 pandemic affected different areas of the study participants’ lives and their experiences related to pregnancy and birth outcomes. In Puerto Rico, 41.7% of the population lives in poverty, and this number rises to 57.6% in the case of young people under 18 years of age [17]. In 2017, the median household income was USD 19,343, approximately one-third of the median household income in the United States (USD 60,336) [18]. According to the results of this study, the pandemic did not affect living conditions significantly, despite more than half of the participants living in poverty according to their annual income. In this sense, we understand that despite the economic assistance from the federal government for COVID-19, this did not change the living conditions of the participants. If anything, it kept them in the same status. For example, many participants expressed that they received economic aid and thus avoided the impact of work situations (i.e., layoffs, reduced hours) and access to food.

In terms of pregnancy and birth experiences, the two situations that affected participants were isolation during and after the birthing process and a change in intended plans for birth and baby showers, for example, physical distancing due to the COVID-19 pre- and post-pregnancy (i.e., baby showers and childcare). This is important to highlight because, in a historical and cultural sense, pregnant women in Puerto Rico expect to give birth accompanied by a person who is considered family. Similarly, the baby shower is a form of support, affection, and a source of material and financial assistance upon the arrival of a baby to a home. The study participants in Puerto Rico reported that 19.8% underwent labor alone, while 38.9% were separated from their newborn after delivery. This is comparable to findings from a study in Indonesia where 13% were denied the presence of a birth companion and 28% of participants reported that their babies had been removed at birth due to protocols or the baby’s health [19].

When considering access to healthcare services during a public health emergency, the most salient issue for the participants was the changes in the birthing process protocol, which resulted in isolation for most of them. These protocols recommended isolation before birth only for suspected or positive cases of COVID-19 to prevent contagion [14]. However, based on participants’ responses, as mentioned in the qualitative interviews, the implementation did not consider this and isolated all pregnant people when they needed support from loved ones, whether to accompany them or to advocate for their rights [14]. The restrictions during the pandemic and, in some cases, the lack of clear explanations due to protocols, had a negative impact on their birth experiences. Social isolation and restrictions in hospitals are some of the factors that affect the mental health and well-being of pregnant and postpartum women and can contribute to the development of depressive symptoms [10].

Another issue that might impact postpartum women’s mental health during the pandemic is related to the many roles and responsibilities that they might assume during this. School and daycare closures and the reduced availability of outside help have led to months of additional work for women. This has meant balancing full-time employment with childcare and schooling responsibilities, particularly for working mothers. In general, the responsibility of caring for sick and elderly family members often falls on women as well [20]. A total of 31.5% of participants in the study mentioned social distancing due to the COVID-19 pandemic as a source of stress. In a study conducted in Louisiana, they found that social distancing (from friends and family and from public places) was associated with anxiety [21]. The most reported sources of stress were regarding their health status and that of their immediate family. Another source of stress was related to their partner’s work situation in most cases and the challenges related to childcare and distance learning.

The most reported coping mechanisms were watching TV or playing video games, followed by using social media and talking with loved ones. In many cases, these coping mechanisms, mainly talking with people they loved and trusted (a strategy to keep the connection between friends and family), seem to have served as an alternative to therapy. Furthermore, regarding mental health and social support, the participants mentioned family support as crucial, specifically the support from their spouses and parents. Badon and collaborators (2022) found similar coping strategies in their study: talking with friends and family, outdoor physical activities, and increasing screen time. They also found that these activities were associated with 29% to 38% less prevalence of moderate/severe depression symptom [22]. Social support is considered a protective factor against depression, and studies have shown an inverse relationship between social support and perinatal depression [23].

In terms of study limitations, we can mention the following: (1) the sample was not representative of the entire Puerto Rican population but was only from a certain geographical region that encompasses several municipalities; (2) there was a lack of verified scales for measuring mental health impact, adverse birth experiences or outcomes; we did not use standardized measures that can be compared across different cohorts; (3) most study participants were not pregnant at the time of survey or interview, and there was no follow-up during the different phases of the pandemic; and (4) recall bias was possible since the surveys were performed after the pregnancy and birth had already passed.

## 5. Conclusions

The findings from this study evidenced that physical distancing as a restriction due to the COVID-19 pandemic was a source of stress to pregnant and postpartum women. This is mostly because they could not carry out activities during pregnancy (like having baby showers), during the birthing process (like having more than one person with them), and during the postpartum period (like receiving visits from immediate family). However, it was the social support, even from a distance, that served as a protective factor to face the multiple challenges related to the pandemic and maternal health.

Finally, regarding future directions, the research team would like to compare experiences during and after Hurricanes Irma and María with those during the COVID-19 pandemic in order to identify common risk and protective factors as well as relevant differences (access to health services). Secondly, the research team thinks that consulting with community experts considering pregnant women, caretakers, healthcare providers, and community organizers could provide relevant insights regarding findings and suggestions for new avenues of research in this population. Finally, the research team would like to use these secondary analyses in consultation with interested parties to inform a new research project regarding maternal health and emergency preparedness.

## Figures and Tables

**Table 1:** Social determinants of health during the COVID-19 pandemic (n = 184).

Socioeconomic and Environmental Factor	Yes		No	
	n	%	n	%
Needed economic assistance during pandemic	88	47.8	96	52.2
Needed food assistance during pandemic	103	56.0	81	44.0
Has public health insurance	115	62.5	69	37.5
Has Wifi access	169	91.8	15	8.2
Family member with COVID-19	64	34.8	120	65.2
Family member died from COVID-19	6	3.3	178	96.7

**Table 2:** Types of social support received (n = 184).

Social Support	Yes		No	
	n	%	n	%
Emotional	46	25.0	138	75.0
Economic	75	40.8	109	59.2
Childcare	63	34.2	121	65.8
Taking care of home	24	13.0	160	87.0
Errands	54	29.3	130	70.7
Medical appointments	42	22.8	142	77.2
Food	49	26.6	135	73.4
Transportation	19	10.3	165	89.7
Other	51	27.7	133	72.3

**Table 3:** Source of social support received (n = 184).

Sources of Social Support	Yes		No	
	n	%	n	%
Partner/Spouse	121	65.8	63	34.2
Family members	151	82.1	33	17.9
Friends	38	20.7	146	79.3
Neighbors	21	11.4	163	88.6
Community organizations	4	2.2	180	97.8
Religious organizations	18	9.8	166	90.2
Government	47	25.5	137	74.5
Other	9	4.9	175	95.1

**Table 4:** Maternal health-related experiences during COVID-19 (n = 131).

Maternal Health-Related Experiences During COVID-19 (n = 131)	Yes		No	
	n	%	n	%
Decided to give birth at home instead of hospital	21	16.0	110	84.0
Decided to give birth at hospital instead of home	54	41.2	77	58.8
Decided to have C-section instead of vaginal birth	30	22.9	101	77.1
Decided to have vaginal birth instead of C-section	39	29.8	92	70.2
Had fewer prenatal appointments	5	3.8	126	96.2
Had more prenatal appointments	16	12.2	115	87.8
Prenatal appointments were virtual	2	1.5	129	98.5
Had to give birth alone	26	19.8	105	80.2
Was separated from newborn after birth	51	38.9	80	61.1
Was in isolation before birth	28	21.4	103	78.6
Was in isolation after birth	26	19.8	105	80.2
None	37	28.2	94	71.8
Other	26	19.8	105	80.2

**Table 5:** COVID-19 prevention measures during pre- and postnatal periods (n = 131).

COVID-19 Prevention Measures During Pre- and Postnatal Periods (n = 131)	Yes		No	
	n	%	n	%
Postpone medical appointments	16	12.2	115	87.8
Cancel medical appointments	16	12.2	115	87.8
Telemedicine	21	16.0	110	84.0
Having food delivered	60	45.8	71	54.2
Ordering food online	26	19.8	105	80.2
Ordering medicine online	10	7.6	121	92.4
Having medicines delivered	9	6.9	122	93.1
Canceling baby shower	33	25.2	98	74.8
Virtual baby shower	7	5.3	124	94.7
Drive-by baby shower	39	29.8	92	70.2
Avoid going out	118	90.1	13	9.9
Avoid going to malls	120	91.6	11	8.4
Avoid visits from people who do not live at home	113	86.3	18	13.7
None	1	0.8	130	99.2
Other	16	12.2	115	87.8

**Table 6:** Sources of stress related to the pandemic (n = 184).

Sources of Stress Related to the Pandemic	Yes		No	
	n	%	n	%
Health status	91	49.5	93	50.5
Work situation	73	39.7	111	60.3
Economic situation	53	28.8	131	71.2
Childcare	54	29.3	130	70.7
Taking care of home	23	12.5	161	87.5
Relationship	19	10.3	165	89.7
Family relationships	28	15.2	156	84.8
Access to health services	27	14.7	157	85.3
Access to education services	27	14.7	157	85.3
Access to food	16	8.7	168	91.3
Access to medicines	9	4.9	175	95.1
Access to baby supplies	30	16.3	154	83.7
Social distancing	58	31.5	126	68.5
COVID19 impact on family	51	27.7	133	72.3
COVID19 impact on community	38	20.7	146	79.3
COVID19 impact on society	48	26.1	136	73.9
Other	22	12.0	162	88.0
None	8	4.3	176	95.7

## Data Availability

The datasets used and/or analyzed during the current study are available from the corresponding author on reasonable request.
